# Supplementary material for: Impact of COVID-19 on the food security and identifying the compromised food security dimension: A systematic review protocol
Source: PLoS One. 2022 Aug 9;17(8):e0272859. doi: 10.1371/journal.pone.0272859 (PMC9362949; doi:10.1371/journal.pone.0272859)
Supplement: Attachment — Submitted filename: Rebuttal Letter (PONE-D-22-13987).pdf [file pone.0272859.s002.pdf]

<u>**Rebuttal Letter to manuscript PONE-D-22-13987**</u>

Dear reviewers and editors

First and foremost, we want to acknowledge the substantial time you spent for reviewing our manuscript entitled "*Impact of COVID-19 on the food security and identifying the compromised food security dimension: A systematic review protocol***"**. The changes in the main manuscript are marked in Microsoft track change and revised, but unmarked manuscript is attached separately.

No	Comments	Responses
	**Reviewer 1**	
1.	L. 26 and other parts of the manuscript; do you want to say google search engine? or google scholar search engine?	The comment is accepted and corrected in the revised manuscript.
2.	L. 26-27: replace the "references of included studies from the databases and manual searches" with studies' reference list were also manually searched.	The comment is accepted and corrected in the revised manuscript.
3.	Please shorten the sentences from line 129-133 the shortest and most informative sentence "The four pillars of food security are interdependent on each other. If one or more of these pillars are affected by COVID-19, the whole food security system will be compromised. Identifying the more affected food security pillars is crucial to intervene in food security alleviation. As a result, all studies that were written on the impact of COVID-19 without considering one or more pillars will be excluded".	The comment is accepted and corrected in the revised manuscript.
4.	L. 148: Who is the owner of the ROBIS tool? Better to include it.	The comment is accepted and corrected in the revised manuscript.
5.	L. 195: Write the AHRQ in full in first use.	The comment is accepted and corrected in the revised manuscript.
	**Reviewer 2**	
1.	Page 2 line 20: "severely" to substantially	The comment is accepted and corrected in the revised manuscript.
2.	Page 2 line 24: "Original research publications" to Primary research focused	The comment is accepted and corrected in the revised manuscript.
3.	Page 4 line 78-79: "Concept" to 'focus', and please paraphrase this sentence in a clear way.	The comment is accepted and corrected in the revised manuscript.
4.	Page 5 line 85: delete "main"	The comment is accepted and corrected in the revised manuscript.
5.	Page 5 line 94: delete "the proposed"	The comment is accepted and corrected in the revised manuscript.
6.	Page 5 line 101: better to delete "original"	The comment is accepted and corrected in the revised manuscript.
7.	Page 5 line 105: better to use the word valuable than "eligible". To say… unincluded and valuable literature.	The comment is accepted and corrected in the revised manuscript.
8.	Page 5 line 109: Please replace the phrase "the senior experts" with 'other team members'	The comment is accepted and corrected in the revised manuscript.
9.	Page 6 line 122: "Redundant" to 'duplicate'	The comment is accepted and corrected in the revised manuscript.
10.	Page 6 line 124: "sort out" to 'removed'	The comment is accepted and corrected in the revised manuscript.

11.	Page 6 line 134: What about qualitative studies?	The comment is accepted and corrected in the revised manuscript.
12.	Page 6 line 135-137: Better to remove the sentence	The comment is accepted and corrected in the revised manuscript.
13.	Page 6 line 138-140: The sentence "To be sure of the content of the literature whether they contain the food security pillars or not, the abstract of the literature will be red before they are excluded as unfit" is repeated.	The comment is accepted and corrected in the revised manuscript.

Daniel Teshome Gebeyehu (corresponding author)
